# Comparison of regional brain atrophy and cognitive impairment between pure akinesia with gait freezing and Richardson's syndrome

**DOI:** 10.3389/fnagi.2015.00180

**Published:** 2015-09-29

**Authors:** Jin Yong Hong, Hyuk Jin Yun, Mun Kyung Sunwoo, Jee Hyun Ham, Jong-Min Lee, Young H. Sohn, Phil Hyu Lee

**Affiliations:** ^1^Department of Neurology, Yonsei University Wonju College of MedicineWonju, South Korea; ^2^Department of Biomedical Engineering, Hanyang UniversitySeoul, South Korea; ^3^Department of Neurology, Bundang Jesaeng General HospitalSeongnam, South Korea; ^4^Department of Neurology and Brain Research Institute, Yonsei University College of MedicineSeoul, South Korea; ^5^Severance Biomedical Science Institute, Yonsei University College of MedicineSeoul, South Korea

**Keywords:** pure akinesia with gait freezing, freezing of gait, progressive supranuclear palsy, cortical thickness, subcortical volume, cognition

## Abstract

Pure akinesia with gait freezing (PAGF) is considered a clinical phenotype of progressive supranuclear palsy. The brain atrophy and cognitive deficits in PAGF are expected to be less prominent than in classical Richardson's syndrome (RS), but this hypothesis has not been explored yet. We reviewed the medical records of 28 patients with probable RS, 19 with PAGF, and 29 healthy controls, and compared cortical thickness, subcortical gray matter volume, and neuropsychological performance among the three groups. Patients with PAGF had thinner cortices in frontal, inferior parietal, and temporal areas compared with controls; however, areas of cortical thinning in PAGF patients were less extensive than those in RS patients. In PAGF patients, hippocampal, and thalamic volumes were also smaller than controls, whereas subcortical gray matter volumes in PAGF and RS patients were comparable. In a comparison of neuropsychological tests, PAGF patients had better cognitive performance in executive function, visual memory, and visuospatial function than RS patients had. These results demonstrate that cognitive impairment, cortical thinning, and subcortical gray matter atrophy in PAGF patients resemble to those in RS patients, though the severity of cortical thinning and cognitive dysfunction is milder. Our results suggest that, PAGF and RS may share same pathology but that it appears to affect a smaller proportion of the cortex in PAGF.

## Introduction

Pure akinesia was first described by Imai et al. in 1974, and their cases were characterized by freezing or blocking during gait, speaking, and writing (Imai and Narabayashi, 1974; Williams et al., 2007). However, patients did not respond to levodopa, nor did they display other characteristics of parkinsonism including limb rigidity, resting tremor, cognitive impairment, or abnormal eye movement. Since this initial description, many similar cases have been reported using various terms including pure akinesia (Imai et al., 1987; Matsuo et al., 1991; Riley et al., 1994), primary progressive freezing of gait (Achiron et al., 1993; Factor et al., 2006), and gait ignition failure (Atchison et al., 1993). Although there are exceptions (Quinn et al., 1989; Imai and Narabayashi, 1990; Molinuevo et al., 2003), most pathological studies consistently suggest that pure akinesia is associated with progressive supranuclear palsy (PSP)-tau pathology (Matsuo et al., 1991; Imai et al., 1993; Mizusawa et al., 1993; Riley et al., 1994; Yoshikawa et al., 1997; Compta et al., 2007; Williams et al., 2007). Williams et al. proposed diagnostic criteria for pure akinesia with gait freezing (PAGF) and selected seven patients fulfilling the criteria from the Queen Square Brain Bank (Williams et al., 2007). Among them, PSP-tau pathology was found in six cases and Lewy body pathology in the other. Therefore, the authors suggested that PAGF should be considered the third phenotype of PSP.

Although, the severity of brain atrophy and cognitive deficit in patients with PAGF is expected to be less prominent than that in patients with classical Richardson's syndrome (RS) by definition, the patterns of brain atrophy or cognitive characteristics of PAGF have not been explored yet. In this study, we compared cortical thickness, subcortical gray matter volume, and cognitive performance between PAGF and RS to investigate the topographic pattern of brain atrophy and cognitive deficits in patients with PAGF.

## Materials and methods

### Participants

The present study was retrospective and enrolled 28 patients with probable RS and 19 from the movement disorders clinic at a tertiary referral center (Yonsei University Severance Hospital) between January 2008 and December 2014. Probable RS was diagnosed according to the NINDS-SPSP clinical criteria (Litvan et al., 1996), and the PAGF were identified using the following criteria proposed by Williams et al.: history of gait or speech freezing of gradual onset, absence of limb rigidity and tremor, no sustained response to levodopa, no dementia or ophthalmoplegia in the first 5 years of disease, and exclusion of vascular disease or Binswanger's disease by magnetic resonance imaging (MRI) (Williams et al., 2007). Parkinsonian motor symptoms were assessed using the Unified PD Rating Scale (UPDRS) part III. All patients were treated with more than 750 mg/day of levodopa, and a patient was regarded as unresponsive to levodopa when the UPDRS part III score reduced less than 30% or increased following at least 6 months of levodopa treatment (Williams et al., 2005). Additionally, 29 healthy age- and sex-matched controls were selected who had completed brain MRI scans and neuropsychological assessment for medical screening. All healthy controls' brains appeared normal on MRI finding, and they displayed normal cognitive performance.

The study protocol was approved by the institutional review board (IRB) on human experimentation, and exempted from providing informed consent by the IRB due to the retrospective design.

### MRI acquisition

A Philips 3.0 T scanner (Philips Achieva; Philips Medical System, Best, the Netherlands) with a SENSE head-8 coil was used to obtain high-resolution T1-weighted magnetic resonance (MR) volume datasets. Imaging was performed using a three-dimensional T1-TFE sequence configured with the following parameters: axial acquisition with a 224 × 224 matrix; 256 × 256 reconstructed matrix; 220 × 220-mm field of view; 0.86 × 0.86 × 1.0-mm voxels; echo time, 4.6 ms; repetition time, 9.6 ms; flip angle, 8°; and slice gap, 0 mm.

### Image processing

#### Cortical thickness

Images were processed using the standard Montreal Neurological Institute (MNI) anatomical pipeline. The native MR images were normalized into a standardized stereotaxic space using affine transformation and intensity non-uniformity artifacts in normalized images were corrected using the N3 algorithm (Collins et al., 1994; Sled et al., 1998). After correction using the brain extraction tool (BET), the skull area was removed from images; then, the remaining areas were classified as white matter (WM), gray matter (GM), cerebrospinal fluid (CSF), or background using an advanced neural net classifier (Smith, 2002). The surfaces of the inner and outer cortex, consisting of 40,962 vertices, were extracted automatically using the Constrained Laplacian-based Automated Segmentation with Proximities (CLASP) algorithm (MacDonald et al., 2000; Kim et al., 2005). Cortical thickness was defined using the t-link method, which captures the Euclidean distance between the linked vertices (MacDonald et al., 2000; Im et al., 2006). Each individual thickness map was transformed to a surface group template using a two-dimensional (2D) surface-based registration after diffusion smoothing with 20-mm full-width half-maximum to increase the signal-to-noise ratio and improve the ability of detection of population changes (Robbins et al., 2004). To assess group differences in cortical thickness, a general linear model was constructed with age, sex, and intracranial volume (ICV) as independent variables and each of the vertices of thickness as a dependent variable. For multiple comparisons, the results were thresholded at a false-discovery-rate corrected *p*-value of 0.05 and cluster of 100 (Genovese et al., 2002).

#### Subcortical gray matter volume

Subcortical structures were automatically segmented in the native space of each MRI using the automated anatomical labeling (AAL) template (Tzourio-Mazoyer et al., 2002). AAL regions were deformed onto a standardized stereotaxic space by inverse spatial normalization. Individual tissue classification maps for GM were used to mask segmentation of native AAL regions. We then computed the volumes of four subcortical regions (hippocampus, caudate, putamen, and thalamus) from the masked individual AAL regions. To compare the volume of subcortical structures between groups, we also applied general linear model with sex, age, and ICV.

### Neuropsychological assessment

The neuropsychological performance of 20 patients with RS and 12 with PAGF was evaluated within 2 months from MRI acquisition, using the following cognitive subsets: forward and backward digit span, the Korean version of the Boston Naming Test (K-BNT), Rey Complex Figure Test (RCFT) copying, 20-min delayed recall using the Seoul Verbal Learning Test and the RCFT, semantic and phonemic fluency using the Controlled Oral Word Association Test (COWAT), and color-word reading of the Stroop test. Mental state was assessed using the Korean version of Mini-Mental State Examination (K-MMSE) and the Clinical Dementia Rating scale (CDR).

### Statistical analysis

For baseline characteristics, categorical variables were compared using the chi-square test, and continuous variables by independent *t*-test and One-Way analysis of variance (ANOVA). Because of the relatively small sample size and skewed distribution of the neuropsychological data, the Kruskal−Wallis test was used to compare neuropsychological performance among groups. For *post hoc* analyses, Bonferroni's method was conducted for ANOVA, and Dunn's test followed by Bonferroni's correction was conducted for the Kruskal−Wallis test. Statistical analyses were performed using SPSS Statistics 20 (IBM SPSS, Armonk, NY, USA), and a *p* < 0.05 was considered to indicate statistical significance.

## Results

### Demographic characteristics

The demographic characteristics and clinical symptoms of the subjects are shown in Table 1. There were no significant differences among RS and PAGF patients, and controls in term of age at MRI scan or gender. RS and PAGF patients were comparable in age at disease onset and time from onset to MRI scan. However, compared with RS patients, the PAGF patients had longer disease duration (8.3 ± 2.5 vs. 4.2 ± 1.9 years, *p* < 0.001) at the time of medical records review and milder motor symptoms (UPDRS motor score; 20.9 ± 9.6 vs. 36.4 ± 16.7, *p* = 0.001) at the first hospital visit. The patients with PAGF had speech disturbance (17.9 vs. 46.4%, *p* = 0.036) and nuchal rigidity (42.1 vs. 89.3%, *p* = 0.001) less frequently than those with RS did, while the frequency of gait disturbance and finger bradykinesia was comparable between the groups of PAGF and RS. No patients with PAGF showed ophthalmoplegia, tremor, or limb rigidity.

**Table 1:** **Demographic characteristics and clinical symptoms of patients with Richardson's syndrome and pure akinesia with gait freezing**.

	**RS**	**PAGF**	**NC**	***P*-value**
Age at MRI, year	67.6 ± 6.5	72.0 ± 6.1	68.4 ± 5.7	0.051
Male/female	14/14	11/8	13/16	0.676
Age at disease onset, year	64.5 ± 7.2	68.1 ± 6.1	N/A	0.082
Disease duration, year	4.2 ± 1.9	8.5 ± 2.7	N/A	< 0.001
Time from onset to MRI, year	3.1 ± 1.8	3.9 ± 2.7	N/A	0.244
Clinical symptom at MRI				
UPDRS motor score	36.4 ± 16.7	20.9 ± 9.6	N/A	0.001
Gait disturbance, n (%)	26 (92.9)	19 (100)	N/A	0.508
Speech disturbance, n (%)	13 (46.4)	15 (17.9)	N/A	0.036
Ophthalmoplegia, n (%)	28 (100)	0 (0)	N/A	< 0.001
Tremor, n (%)	6 (21.4)	0 (0)	N/A	0.068
Finger bradykinesia, n (%)	12 (42.9)	3 (15.8)	N/A	0.063
Limb rigidity, n (%)	22 (78.6)	0 (0)	N/A	< 0.001
Nuchal rigidity, n (%)	25 (89.3)	8 (42.1)	N/A	0.001

*Data are expressed as mean ± SD or n (%)*.

*RS, Richardson's syndrome; PAGF, pure akinesia with gait freezing; NC, normal control; MRI, magnetic resonance imaging; UPDRS, Unified Parkinson's disease rating scale; N/A, not applicable*.

### Cortical thickness

**Figure 1:**
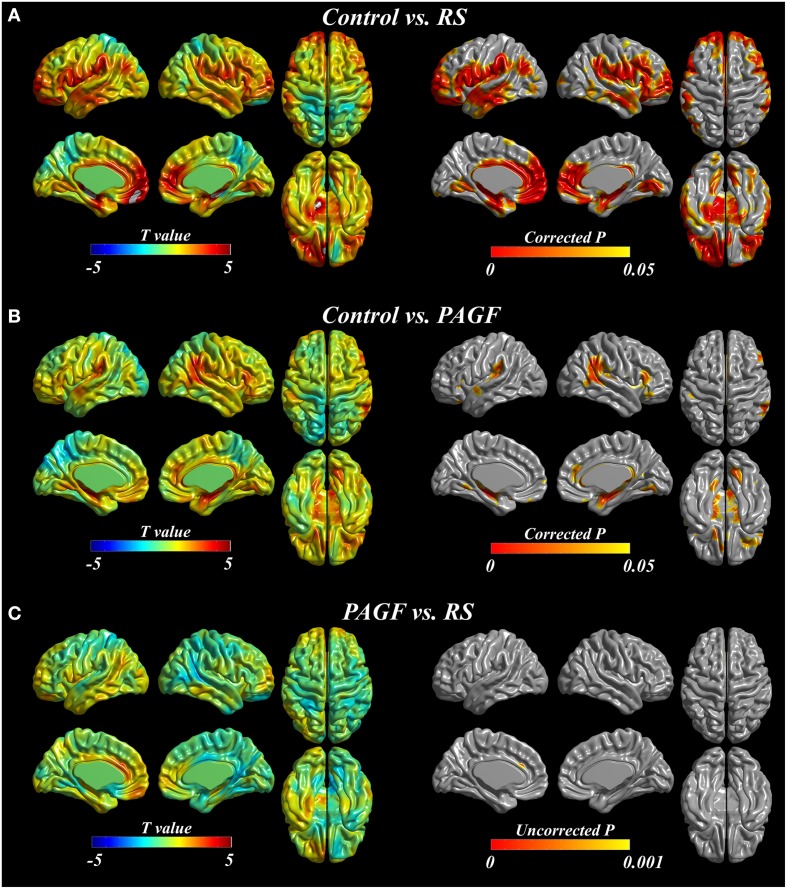
**Cortical thickness analyses in patients with Richardson's syndrome (RS), pure akinesia with gait freezing (PAGF), and controls**. Comparisons of **(A)** RS patients with controls, **(B)** PAGF patients with controls, and **(C)** RS patients to PAGF patients.

The results of cortical thickness analyses are shown in Figure 1. PAGF patients had significant cortical thinning in bilateral inferior frontal, left orbitofrontal, right anterior cingulate, bilateral insula, bilateral supramarginal, right angular and precunial, and bilateral temporal areas, extending into the parahippocampal and lingual gyri (Figure 1B, Electronic Supplementary Table 1). Areas where the cortex was thinner in RS patients compared with controls were extensive, involving bilateral frontal, bilateral anterior and middle cingulate, bilateral insula, bilateral pericentral, bilateral parietal (bilateral supramarginal and left angular and inferior parietal areas), and bilateral temporal areas, extending into parahippocampal and occipital areas (Figure 1A, Electronic Supplementary Table 2). Cortical thickness did not significantly differ between patients with PAGF and those with RS. When compared at a liberal threshold (uncorrected *p* < 0.001), the cortex of RS was thinner in the anterior cingulate gyrus compared with the PAGF patients (Figure 1C, Electronic Supplementary Table 3). No areas where PAGF patients exhibited more cortical thinning than RS patients were observed.

### Subcortical gray matter volume

Compared with controls, PAGF patients had smaller volume in left hippocampus (*post hoc p* = 0.005) and left thalamus (*post hoc p* = 0.018), whereas there was no volume loss in caudate nucleus and putamen (Table 2). Similarly, RS patients also had smaller volume in left hippocampus (*post hoc p* = 0.014) and bilateral thalami (*post hoc p* = 0.015 for right, 0.024 for left) than did controls. There was no volume difference between PAGF and RS patients.

**Table 2:** **Volumetric analyses for subcortical gray matter in patients with Richardson's syndrome and pure akinesia with gait freezing**.

	**RS**	**PAGF**	**NC**	***P*-value**	***Post hoc P*****-value**		
					**RS vs. NC**	**PAGF vs. NC**	**RS vs. PAGF**
Hippocampus	8251 ± 699	8222 ± 607	8635 ± 780	0.020	ns	0.038	ns
Right	4102 ± 383	4100 ± 386	4241 ± 401	ns			
Left	4148 ± 380	4122 ± 298	4394 ± 408	0.002	0.014	0.005	ns
Caudate nucleus	7759 ± 1382	7965 ± 1185	7750 ± 936	ns			
Right	4118 ± 826	4252 ± 650	4007 ± 436	ns			
Left	3641 ± 607	3712 ± 575	3743 ± 522	ns			
Putamen	6620 ± 1455	6373 ± 1459	6319 ± 1663	ns			
Right	3556 ± 777	3374 ± 712	3365 ± 865	ns			
Left	3063 ± 711	3000 ± 764	2954 ± 854	ns			
Thalamus	7277 ± 1205	7488 ± 1385	8038 ± 1499	0.006	0.012	0.028	ns
Right	3617 ± 681	3792 ± 771	4021 ± 790	0.011	0.015	ns	ns
Left	3660 ± 567	3697 ± 690	4017 ± 769	0.007	0.024	0.018	ns

*Data are expressed as mean ± SD*.

*RS, Richardson's syndrome; PAGF, pure akinesia with gait freezing; NC, normal control; ns, not significant*.

### Neuropsychological findings

The neuropsychological performance of the RS, PAGF, and control groups is presented in Table 3. The duration of education was comparable among the three groups. K-MMSE scores of RS and PAGF patients were significantly lower than those of controls, but there was no difference between the two patient groups.

**Table 3:** **Neuropsychological performance of patients with Richardson's syndrome and pure akinesia with gait freezing**.

	**RS (*****n*** = 20**)**		**PAGF (*****n*** = 12**)**		**NC (*****n*** = 29**)**		***P*-value**	***Post hoc P*****-value**		
	**median**	**IQR**	**median**	**IQR**	**median**	**IQR**		**RS vs. NC**	**PAGF vs. NC**	**RS vs. PAGF**
Years of education, year	9	7.5-12	13.5	11.5−16	14	8−16	0.196			
K-MMSE score	23.5	17−26.5	26	25−27.5	29	28−29	< 0.0001	< 0.0001	0.002	ns
CDR	0.75	0.5−1	0.5	0.25−0.5	0	0−0.5	< 0.0001	< 0.0001	0.03	ns
Neuropsychological tests										
Forward digit span	5.5	4−6	6	5−6.5	6	6−7	0.004	0.005	ns	ns
Backward digit span	2	0−3	3.5	2.5−4	4	4−4	< 0.0001	< 0.0001	ns	ns
K-BNT	39.5	26.5−43.5	42	38−49	50	46−53	< 0.0001	< 0.0001	0.03	ns
RCFT copy	18.5	9−26.5	33.5	30−34	36	35−36	< 0.0001	< 0.0001	0.04	0.02
SVLT delayed recall	2.5	0.5−4	3.5	2−4.5	7	6−8	< 0.0001	< 0.0001	0.0006	ns
RCFT delayed recall	6	2−10	15.5	10.5−19	18.5	15.5−24	< 0.0001	< 0.0001	ns	0.009
Semantic fluency	11	8−17	27	21−31.5	39	33−44	< 0.0001	< 0.0001	0.03	0.02
Phonemic fluency	5	3−10.5	19.5	13.5−29.5	26	21−30	< 0.0001	< 0.0001	ns	0.002
Color-word Stroop	20	5.5−30.5	63.5	48.5−80.5	89.5	78.5−109	< 0.0001	< 0.0001	ns	0.005

*RS, Richardson's syndrome; PAGF, pure akinesia with gait freezing; NC, normal control; IQR, interquartile range; K-MMSE, Korean version of Mini-mental state examination; CDR, clinical dementia rating; K-BNT, Korean version of Boston Naming Test; RCFT, Rey complex figure test; SVLT, Seoul verbal learning test; ns, not significant*.

*The analysis was performed using the analysis of covariance with age and years of education as covariates. Post hoc analyses were conducted using Dunn's methods with Bonferroni's correction*.

RS patients showed poorer performance on all tested neuropsychological subsets compared with controls, including attention (forward and backward digit span), confrontational naming (K-BNT), visuospatial function (RCFT copy), verbal and visual memory (delayed recall of SVLT and RCFT), verbal fluency (both semantic and phonemic tasks), and color-word Stroop test. PAGF patients had significantly poorer scores in confrontational naming, the visuospatial task, verbal memory, and semantic fluency compared with controls. When cognitive scores of PAGF and RS patients were compared, PAGF patients scored higher on the visuospatial task, visuospatial memory, both semantic and phonemic fluencies, and the color-word Stroop test.

## Discussion

This is the first study investigating cognitive performance and brain atrophic pattern in PAGF. PAGF patients had cortical thinning in frontal, inferior parietal, and temporal areas compared with controls; however, the areas of cortical thinning in patients with PAGF were less extensive than in RS patients. Additionally, PAGF patients had smaller volume in the hippocampus and thalamus than controls had, whereas their subcortical gray matter volumes were comparable to those in RS patients. In a comparison of neuropsychological tests, PAGF patients had better cognitive performance in frontal executive function, visual memory, and visuospatial function than did RS patients. These data suggest that PAGF may involve a similar pathological burden in subcortical gray matter as in RS but that this pathology may occur in a more limited cortical area than in RS, which may underlie the difference in cognitive performance.

Previous pathological studies indicate that the distribution and severity of pathological changes in PAGF are milder than in RS. Specifically, PAGF involves less severe tau pathology in the motor cortex, striatum, pontine nuclei, and cerebellum than in RS (Williams et al., 2007; Ahmed et al., 2008). These results suggest that the difference in pathological burden between the two diseases may explain the relatively milder clinical symptoms of PAGF. Moreover, a functional neuroimaging study using positron emission tomography also revealed that although cerebral metabolism is similar, PAGF patients displayed an attenuated pattern of brain glucose metabolism with preservation of frontal metabolism compared with RS patients (Park et al., 2009). In the present study, cortical thickness analysis indicated that PAGF has cortical thinning in inferior and medial frontal, inferior parietal, and temporal areas, whereas superior frontal, superior parietal, and occipital areas are preserved. Interestingly, the same areas are thinned in RS patients, but in our RS patients, cortical thinning affected a more extensive proportion of the brain, including inferior and medial frontal, parietal, and temporal areas, consistent with previous MR-based volumetric neuroimaging studies (Brenneis et al., 2004; Cordato et al., 2005). Direct comparison indicates that PAGF involves greater cortical thinning in the anterior cingulate gyrus than that seen in RS. Therefore, the present results are in line with pathological studies in supporting the conclusion that PAGF shares pathological characteristics with RS, but to a less severe extent.

Subcortical gray matter volume analysis revealed that PAGF patients have significant volume loss in left hippocampus and left thalamus compared with controls; the distribution of volume loss in subcortical gray matter was quite similar to that in RS. Further direct comparison showed that subcortical volume loss in these areas was comparable between PAGF and RS patients. Previous imaging studies have found diverse patterns of subcortical gray matter volume loss in RS; several investigators reported general atrophy in wide subcortical areas including the hippocampus, caudate nuclei, globus pallidus, putamen, and thalamus, while others did not detect any significant volume loss in these areas (Schulz et al., 1999; Cordato et al., 2000, 2002, 2005; Hanyu et al., 2001; Brenneis et al., 2004; Paviour et al., 2006; Messina et al., 2011). To our knowledge, ours in the first direct comparison of subcortical structures between PAGF and RS, finding that the severity and distribution of subcortical gray matter pathology are similar. However, a further study with a larger sample and more broadly accepted methodology is needed to resolve whether the subcortical pathological burden differs between PAGF and RS.

Cognitive impairment is well-known to be common in RS; about two-thirds of RS patients exhibit below-normal cognitive performance (Rittman et al., 2013). The most common deficit in RS is frontal executive dysfunction, which appears early in disease progression (Pillon et al., 1986; Soliveri et al., 2000; Lange et al., 2003; Bak et al., 2005; Rittman et al., 2013). Moreover, phonemic and semantic verbal fluency are severely impaired in RS, a key differentiator from Parkinson's disease or corticobasal degeneration (Pillon et al., 1991; Rittman et al., 2013). Additionally, about one-third of PSP patients also exhibit dysfunction in other cognitive domains, such as memory and visuospatial function (Pillon et al., 1986; Soliveri et al., 2000; Lange et al., 2003; Bak et al., 2005). However, no characteristic pattern of cognitive impairment has been determined in PAGF. In the present study, relative to controls, RS patients showed poorer cognitive performance in attention, naming, the color-word Stroop test, semantic and phonemic verbal fluencies, memory, and visuospatial tasks, in accordance with previous neuropsychological results. Cognitive dysfunction in PAGF appeared less extensive than in RS; PAGF patients had lower scores in semantic fluency, naming, verbal memory, and visuospatial function tasks than controls. Directly comparing the cognitive performance of PAGF with that of RS patients revealed better scores in executive function, visual memory, and visuospatial function tasks in PAGF patients. To speculate on the neural correlates of cognitive dysfunction in patients with PAGF, lower semantic fluency may result from cortical thinning in frontal areas, impairment of verbal memory may be related to hippocampal atrophy or cortical thinning in the parahippocampal gyrus, and visuospatial dysfunction could be associated with posterior cortical thinning. On the other hand, better cognitive performance in PAGF compared with RS patients may result from the difference in the extent of areas with cortical thinning rather a difference in subcortical volume loss, as subcortical gray matter volume loss was comparable. These findings additionally support the conclusion that the cortical pathological burden is reduced in PAGF compared with RS patients, as this would lead to better cognitive performance.

This study has several limitations. First, the clinical diagnoses of study subjects were not confirmed by pathological examination. Especially in PAGF, this leaves open the possibility that the sample included patients with a related disease, such as PD-like pathology (Williams et al., 2007). Second, although this study enrolled the largest number of PAGF patients until now, it is still a relatively small sample. Further studies with larger samples are required to confirm the present results. Finally, the uncorrected threshold used in direct comparison between cortical thickness of PAGF and RS patients may not fully protect against results due to chance, increasing the likelihood of false positives. Therefore, the significant clusters found in the present study require further validation.

In conclusion, this study demonstrates that cognitive impairment, cortical thinning, and subcortical gray matter atrophy in PAGF resemble to RS, but that the severity of cortical thinning and cognitive dysfunction is milder in PAGF. These results suggest that PAGF and RS may share pathology, but its extent and distribution may be less in PAGF than in RS.

## Funding

This study was supported by a grant of the Korea Health Technology R&D Project through the Korea Health Industry Development Institute (KHIDI) funded by the Ministry of Health & Welfare, Republic of Korea (grant number: HI14C0093), and by the National Research Foundation of Korea (NRF) grant funded by the Korea government (MEST) (grant number: 2011-0028333).

### Conflict of interest statement

The authors declare that the research was conducted in the absence of any commercial or financial relationships that could be construed as a potential conflict of interest.
