# Supplementary material for: Comparison of regional brain atrophy and cognitive impairment between pure akinesia with gait freezing and Richardson's syndrome
Source: Front Aging Neurosci. 2015 Sep 29;7:180. doi: 10.3389/fnagi.2015.00180 (PMC4586277; doi:10.3389/fnagi.2015.00180)
Supplement: Supplementary file 1 [file Table1.DOCX]

**Supplementary Table 1**. Cortical thinning in patients with pure akinesia with gait freezing compared with controls.

| Region | Side | Cluster size | Peak T value | MNI coordinate | | |
| --- | --- | --- | --- | --- | --- | --- |
|  |  |  |  | x | y | z |
| superior frontal gyrus, orbital part | Left | 197 | 3.77 | -12.83 | 20.92 | -25.46 |
| inferior frontal gyrus, pars orbitalis | Right | 319 | 3.25 | 32.14 | 34.51 | -13.48 |
| Inferior frontal gyrus, pars triangularis | Left | 172 | 3.89 | -31.97 | 29.66 | 0.52 |
|  | Right | 340 | 3.78 | 52.90 | 30.59 | 14.61 |
| anterior cingulate gyrus | Right | 293 | 3.29 | 11.26 | 40.52 | 13.97 |
| gyrus rectus | Left | 127 | 3.75 | -6.04 | 42.54 | -26.23 |
| insula | Left | 110 | 3.70 | -30.42 | 28.37 | -0.58 |
|  | Right | 164 | 3.68 | 30.59 | 28.90 | 3.44 |
| supramarginal gyrus | Left | 292 | 4.07 | -54.63 | -31.09 | 26.42 |
|  | Right | 384 | 4.24 | 55.55 | -46.04 | 34.77 |
| angular gyrus | Right | 133 | 4.80 | 58.88 | -51.23 | 34.66 |
| precuneus | Right | 201 | 3.82 | 9.78 | -42.40 | 5.75 |
| transverse temporal gyrus | Left | 190 | 4.05 | -40.80 | -20.76 | 4.78 |
|  | Right | 169 | 3.86 | 44.54 | -16.48 | 4.44 |
| superior temporal gyrus | Left | 414 | 4.03 | -41.94 | -20.30 | 2.32 |
|  | Right | 875 | 4.73 | 45.50 | -15.84 | -0.35 |
| superior temporal pole | Right | 159 | 4.11 | 27.63 | 0.22 | -24.85 |
| middle temporal gyrus | Right | 663 | 4.71 | 51.78 | -47.53 | 9.53 |
| parahippocampal gyrus | Left | 188 | 5.61 | -35.99 | -26.05 | -13.75 |
|  | Right | 194 | 4.26 | 32.73 | -9.80 | -24.64 |
| lingual gyrus | Left | 115 | 3.51 | -15.82 | -51.83 | -7.65 |
|  | Right | 229 | 3.80 | 11.17 | -70.55 | -8.53 |
