# Supplementary material for: Comparison of regional brain atrophy and cognitive impairment between pure akinesia with gait freezing and Richardson's syndrome
Source: Front Aging Neurosci. 2015 Sep 29;7:180. doi: 10.3389/fnagi.2015.00180 (PMC4586277; doi:10.3389/fnagi.2015.00180)
Supplement: Supplementary file 2 [file Table2.DOCX]

**Supplementary Table 2**. Cortical thinning in patients with Richardson’s syndrome compared with controls.

| Region | Side | Cluster size | Peak T value | MNI coordinate | | |
| --- | --- | --- | --- | --- | --- | --- |
|  |  |  |  | x | y | z |
| superior frontal gyrus, dorsolateral | Left | 690 | 5.04 | -24.39 | 53.73 | -0.56 |
|  | Right | 492 | 5.24 | 28.19 | 55.75 | 7.85 |
| superior frontal gyrus, medial part | Left | 1123 | 5.53 | -8.73 | 56.53 | -1.58 |
|  | Right | 454 | 4.60 | 12.70 | 49.89 | -2.26 |
| superior frontal gyrus, medial orbital part | Left | 408 | 6.66 | -6.86 | 51.94 | -12.10 |
|  | Right | 365 | 4.34 | 10.41 | 49.03 | -6.29 |
| superior frontal gyrus, orbital part | Left | 867 | 5.47 | -24.85 | 56.41 | -4.10 |
|  | Right | 250 | 4.37 | 27.43 | 52.08 | 0.27 |
| middle frontal gyrus, lateral part | Left | 570 | 5.09 | -29.14 | 53.14 | -1.24 |
|  | Right | 1036 | 5.06 | 27.69 | 53.27 | 7.36 |
| middle frontal gyrus, orbital part | Left | 350 | 5.24 | -28.63 | 54.18 | -2.75 |
|  | Right | 344 | 3.73 | 30.76 | 54.15 | -0.73 |
| inferior frontal gyrus, pars opercularis | Left | 520 | 5.04 | -47.36 | 8.49 | 17.41 |
|  | Right | 427 | 4.18 | 47.36 | 8.49 | 17.41 |
| [inferior frontal gyrus](http://neuro.imm.dtu.dk/services/brededatabase/WOROI_681.html), pars orbitalis | Left | 616 | 4.59 | -32.28 | 28.40 | -3.08 |
|  | Right | 852 | 4.65 | 33.18 | 28.16 | -3.86 |
| Inferior frontal gyrus, pars triangularis | Left | 750 | 5.14 | -39.16 | 23.43 | 6.96 |
|  | Right | 773 | 5.24 | 38.44 | 29.34 | 2.16 |
| gyrus rectus | Left | 363 | 7.27 | -3.21 | 44.68 | -26.99 |
|  | Right | 178 | 3.89 | 3.88 | 28.50 | -28.21 |
| anterior cingulate gyrus | Left | 627 | 4.75 | -11.92 | 45.54 | -6.05 |
|  | Right | 868 | 5.74 | 13.24 | 42.86 | 13.95 |
| middle cingulate gyrus | Left | 406 | 4.00 | -2.49 | 12.30 | 32.08 |
|  | Right | 167 | 3.74 | 10.61 | 29.96 | 28.00 |
| precentral gyrus | Left | 370 | 5.14 | -47.75 | 7.18 | 16.80 |
|  | Right | 413 | 4.40 | 60.12 | -2.29 | 17.95 |
| postcentral gyrus | Left | 325 | 5.38 | -48.87 | -10.60 | 16.10 |
|  | Right | 360 | 4.36 | 61.00 | -4.07 | 15.78 |
| rolandic operculum | Left | 445 | 5.40 | -46.20 | -10.64 | 15.75 |
|  | Right | 395 | 4.40 | 40.79 | -13.68 | 20.24 |
| insula | Left | 701 | 4.85 | -38.42 | -14.65 | -5.05 |
|  | Right | 734 | 4.38 | 39.87 | -2.70 | -13.82 |
| supramarginal gyrus | Left | 564 | 4.82 | -57.12 | -33.06 | 30.36 |
|  | Right | 668 | 3.97 | 61.19 | -39.92 | 25.84 |
| angular gyrus | Left | 356 | 4.25 | -48.61 | -69.69 | 27.90 |
| inferior parietal lobule | Left | 239 | 4.29 | -60.86 | -32.83 | 41.23 |
| cuneus | Left | 155 | 3.09 | -22.00 | -52.39 | 0.35 |
| transverse temporal gyrus | Left | 239 | 4.41 | -39.69 | -22.58 | 2.26 |
|  | Right | 248 | 3.80 | 43.44 | -17.90 | 4.05 |
| superior temporal gyrus | Left | 1122 | 6.70 | -42.97 | -9.17 | -10.48 |
|  | Right | 997 | 5.57 | 44.63 | 0.26 | -14.92 |
| superior temporal pole | Left | 435 | 5.97 | -43.25 | 0.97 | -17.66 |
|  | Right | 442 | 5.25 | 44.17 | 1.78 | -16.69 |
| middle temporal gyrus | Left | 1308 | 3.87 | -62.99 | -22.48 | -20.29 |
|  | Right | 756 | 3.83 | 61.81 | -1.35 | -24.41 |
| inferior temporal gyrus | Left | 508 | 3.92 | -60.88 | -26.45 | -21.82 |
|  | Right | 185 | 2.79 | 64.56 | -30.19 | -13.21 |
| parahippocampal gyrus | Left | 664 | 7.51 | -36.49 | -27.75 | -12.58 |
|  | Right | 390 | 6.01 | 35.64 | -18.99 | -17.68 |
| fusiform gyrus | Left | 134 | 3.78 | -29.26 | -5.55 | -35.81 |
|  | Right | 214 | 2.72 | 38.22 | -74.64 | -14.46 |
| lingual gyrus | Left | 559 | 3.15 | -21.10 | -52.78 | 0.11 |
|  | Right | 394 | 3.87 | 8.46 | -67.17 | -0.32 |
| calcarine sulcus | Left | 167 | 2.77 | -7.03 | -73.22 | 0.49 |
|  | Right | 360 | 3.62 | 5.03 | -77.04 | -1.31 |
| superior occipital gyrus | Right | 105 | 2.77 | 23.69 | -98.18 | 8.11 |
| [middle occipital](http://neuro.imm.dtu.dk/services/brededatabase/WOROI_176.html) gyrus | Left | 496 | 4.26 | -46.98 | -69.98 | 27.80 |
