# Supplementary material for: Comparison of regional brain atrophy and cognitive impairment between pure akinesia with gait freezing and Richardson's syndrome
Source: Front Aging Neurosci. 2015 Sep 29;7:180. doi: 10.3389/fnagi.2015.00180 (PMC4586277; doi:10.3389/fnagi.2015.00180)
Supplement: Supplementary file 3 [file Table3.DOCX]

**Supplementary Table 3**. Cortical thinning in patients with Richardson’s syndrome compared with pure akinesia with gait freezing.

| Region | Side | Cluster size | Peak T value | MNI coordinate | | |
| --- | --- | --- | --- | --- | --- | --- |
|  |  |  |  | x | y | z |
| Anterior cingulate gyrus | Left | 87 | 3.67 | -5.95 | 30.25 | 24.17 |
